# Using primary literature on SARS‐CoV‐2 to promote student learning about evolution

**DOI:** 10.1002/ece3.6501

**Published:** 2020-07-08

**Authors:** Jeremy L. Hsu

**Affiliations:** ^1^ Schmid College of Science and Technology Chapman University Orange California USA

**Keywords:** primary literature, SARS‐CoV‐2 evolution, teaching of evolution

## Abstract

The ongoing COVID‐19 pandemic caused by SARS‐CoV‐2 has caused widespread deaths, illnesses, and societal disruption. I describe here how I pivoted a discussion‐based senior biology capstone course to include a multiweek module surrounding one primary literature paper on the evolution of SARS‐CoV‐2 and the subsequent scientific discourse about the paper. Using a gradual reveal of the paper following the CREATE method (consider, read, elucidate, and think of the next experiment), I challenged students to learn new evolutionary principles and critically analyze the data surrounding the evolution and transmission of SARS‐CoV‐2 presented in the paper. I also provide general advice for implementing this module in future courses.

## 1 INTRODUCTION

The ongoing COVID‐19 pandemic is caused by SARS‐CoV‐2, a novel coronavirus that most likely originated near Wuhan, China, from an animal host (Li et al., 2020). The virus has infected over three million people and caused nearly a quarter‐million deaths worldwide (as of 1 May 2020), while also leading to severe societal disruption, including the widespread transition of colleges and universities to online learning. Concomitant with the pandemic has been an increase in public discourse and speculation about the evolution and origin of the virus, despite numerous studies examining its evolution and zoonotic origin (e.g., Lam et al., 2020; Li et al., 2020).

Given this widespread discourse about the evolution of SARS‐CoV‐2 and the immediate relevance of this topic amid the ongoing pandemic, I decided to modify my senior biology capstone course already underway in the spring semester of 2020 to incorporate a module on SARS‐CoV‐2 evolution. Here, I describe how I pivoted this course using primary literature following the CREATE (consider, read, elucidate, and think of the next experiment) model, discuss the challenges and lessons learned from conducting such a class with online instruction, and provide advice for faculty looking to incorporate this module in future classes.

## 2 BACKGROUND ON COURSE

The senior capstone course was underway in the spring semester of 2020 at a comprehensive, R2 university located in southern California, when the pandemic was declared in March 2020. 17 students were enrolled in the in‐person course in the spring, which is required of (and only open to) senior biology majors. None of these 17 students had previously taken an upper‐level evolution course, meaning that their only formal evolution background at the university was in the introductory biology sequence that most biology students take in their first year.

The course follows a unique format—the program prescribes the course learning outcomes as well as the use of the CREATE (consider, read, elucidate, and think of the next experiment) model (see section below). The learning outcomes are listed below:

1. Students will demonstrate scholarly thinking characteristic of a working biologist.
2. Students will devise and defend a proposed experiment.
3. Students will critically evaluate the relative value of different experiments.
4. Students will think about the broader significance of data.
5. Students will demonstrate strong written and verbal communication skills.

To support these learning outcomes, faculty teaching the course can choose a series of four primary research papers relating to one topic of their choice and center the course on reading and discussing these papers. Students are then challenged to write a four‐page NSF grant proposal and present their ideas at the end of the course. I taught one section of this course in fall 2019 and chose the evolution of gene expression as the central topic, while for the spring 2020 semester I had chosen speciation as the theme. I then carefully chose four papers about speciation (Chamberlain, Hill, Kapan, Gilbert, & Kronforst, 2009; Davidson & Balakrishnan, 2016; Irwin, Bensch, & Price, 2001; Rudman & Schluter, 2016) that would provide exposure to a range of different evolutionary topics and techniques relating to studying speciation.

## 3 THE CREATE METHOD

The course follows the CREATE (consider, read, elucidate, and think of the next experiment) model, a published pedagogical technique for guiding students through reading primary literature that has been shown to increase student understanding and comprehension of the papers (Gottesman & Hoskins, 2013; Hoskins, Lopatto, & Stevens, 2011; Stevens & Hoskins, 2014). The class was structured around this CREATE method and a gradual reveal of each of the four papers, where students read a different section of each paper for each class and complete an assignment designed to guide students to think critically about the content. These readings and assignments are described briefly here:

- Students first read the introduction and generate a concept map of the key terms and concepts. When making this concept map, students must first generate a list of key ideas and terms used in the introduction, and then form connections between these terms by using explicit linking verbs or phrases. Concept maps have been shown to promote deeper learning by facilitating connections between otherwise disparate ideas and have been shown to lead to deeper learning of evolutionary principles (e.g., Apodaca, McInerney, Sala, Katinas, & Crisci, 2019; Schwendimann & Linn, 2015; Stewart, Van Kirk, & Rowell, 1979). In class, I often task each group with generating a consensus concept map that merges the ideas and terms from each student, or ask students to incorporate and connect additional terms and concepts in this consensus concept map from past papers.
- Students next read the methods and draw a cartoon of the methods. This approach is intended to provide students the opportunity to visualize and simplify complex methods. Students are tasked with sketching out the main parts of the experiment, and thus, this assignment allows students to focus on understanding these key experimental methods. In class, I often assign each group one part of the experimental methods to present on and summarize for the class.
- Students next are given the figures and their corresponding captions, and annotate those figures. Annotation involves taking notes on the figure, defining labels, and summarizing key themes from the figures. Similarly to the previous class, I have each group present on one figure or one panel of a figure; students share their annotated figures and use this as a platform to lead the discussion on each figure.
- Students next read the results and are asked to bullet point the results and come up with a proposed discussion of their own. This two‐part assignment thus serves two purposes—first, it allows students the chance to summarize the main findings of the results in their own words. Second, it emulates the scientific process by challenging students to critically think and interpret what these results mean in the context of the paper. I instruct students to think about how they would frame their own discussion section if they had been the scientists conducting these experiments, and ask them to think about what their main findings and conclusions would be.
- Students then read the paper's discussion and compare and contrast their proposed discussion (from the last assignment) with the actual discussion. Students are given the opportunity to see how their proposed discussion compares to the actual one, and this forms the foundation of the in‐class discussion. Students are also tasked to come up with a list of follow‐up questions they have after reading the paper.
- At the end of each paper, students use the list of follow‐up questions they have generated to think of a proposed next experiment to build upon the findings of the paper and outline this proposed next experiment, including the question, hypotheses, predictions, and experimental overview. This again emulates the scientific process by having students think critically and generate new questions and ideas.

Each paper thus takes approximately 3 weeks of class time, with each of the twice weekly 75‐min classes dedicated to discussion of one of these sections (i.e., introduction, materials and methods, figures, results, discussion, and proposed next step) and assignments.

## 4 PIVOTING THE COURSE AND CHOOSING NEW PAPERS

We had finished discussing the first paper on speciation and had begun the second paper on this topic when the COVID‐19 outbreak began to escalate, particularly in the United States. I proposed switching the topic for the last two papers to SARS‐CoV‐2, to which the students readily agreed. While changing topics midway through the semester was challenging, I felt that I could still support the course's learning outcomes given that students would still be reading, discussing, and critically analyzing primary literature, and that I would maintain a focus on evolutionary biology by having at least one of the two remaining papers that students would read in the course center on the evolution of SARS‐CoV‐2. There are a plethora of published papers and preprints on SARS‐CoV‐2, and I decided to choose two contrasting papers focusing on different aspects of SARS‐CoV‐2. As such, I chose a more molecular biology‐oriented study for the first paper; this article describes a series of experiments elucidating the mechanism of SARS‐CoV‐2 entry into host cells (Hoffman et al., 2020). In addition, this paper included a phylogenetic analysis of the spike protein sequence of SARS‐CoV‐2 and other coronaviruses, thus allowing me to introduce and discuss the relevance of phylogenetic analyses and the inferences that can be made from such phylogenetic trees.

For the second paper, I chose a publication that examined the evolution and spread of SARS‐CoV‐2; the authors of this paper conducted population genetics analyses on over 100 published SARS‐CoV‐2 genomes and concluded that there are two unique “types” of the virus with distinct haplotypes, as well as that there are signs of both negative and positive selection in the viral genomes (Tang et al., 2020). I chose this paper for several reasons. First, the paper centers on several key population genetics concepts, ideas, and tests, including searching for signatures of selection with dN/dS ratios and site frequency spectra, looking at rates of recombination across pairs of single nucleotide polymorphisms, and analyzing haplotype networks. Second, the paper also conducts phylogenetic analyses, allowing us to build upon the discussion of phylogenies from the previous paper. Finally, there has been a large amount of scientific discourse surrounding this paper, including calls for its retraction and a correction made by the authors (e.g., see Maclean, Orton, Singer, & Robertson, 2020). This rich scientific discourse and debate centering on both the methods and the analyses used in the paper and the conclusions drawn from the results allowed the opportunity for students to read scientific discourse in nearly real time, given the recency of the paper and the dialog about the manuscript, and also provided a forum for me to challenge students to critically think about the methods, results, and interpretations presented in the paper and draw their own conclusions.

## 5 SUMMARY OF EXPERIENCES

While the course is ongoing as of the time of submission, I have completed discussion of Hoffman et al. (2020) and have nearly completed that of Tang et al. (2020). Instruction was impacted prior to beginning the discussion of Hoffman et al. (2020) when our university closed our campus and transitioned to online learning. Despite this, I was able to continue with this SARS‐CoV‐2 module as planned through using Zoom and other online platforms to continue the course synchronously. Each 75‐min class was structured in a similar manner, where I first highlighted a few key themes, before splitting the class into groups of 3–4 students each at random using Zoom's “breakout rooms” feature. Students would then be given between 15 and 30 min to discuss the reading and their assignment and given prompts about the paper in their small groups, and I would check in on the groups during this time to clarify any points of confusion and answer questions. The final half of class would be spent with a whole‐class discussion of the key concepts and ideas presented in the readings; depending on the pace and content of the day's reading, sometimes I would ask each group to lead the discussion on a given section of the reading. I followed the CREATE model and modified it by adding a class lesson centered on discussing the scientific discourse about the paper; students read a series of messages from other groups and the authors' responses, as well as some outside scientific perspectives on the paper (Maclean et al., 2020; Science Media Centre, 2020). Included in this thread were calls for the paper's retraction and dispute over the paper's methods, analyses, and interpretations, which led to the authors submitting a correction for their paper.

While I have not collected any data, I have anecdotally found that student engagement has increased as compared to our discussions of speciation and that student interest has been piqued in these key evolutionary topics given the relevant context that they are presented in. I have also been pleased to see students embracing these ideas in their final projects, where they write four‐page NSF grants based upon these ideas, and the deep evolutionary thought that discussing these papers has sparked in students.

## 6 ADVICE FOR IMPLEMENTATION OF THIS MODULE

I provide here some general perspectives and advice for faculty wishing to follow a similar model in their courses.

- **Scaffold papers to start from less complex papers**—I have found that starting with more straightforward papers that are more accessible to undergraduate students before scaffolding to more complex papers builds student confidence and provides them the framework needed to grasp the more complex papers. In addition, this can help with breaking down more complex methodology; for example, my students benefitted from discussing phylogenetic analyses first in Hoffman et al. (2020) before diving deeper into phylogenetic analyses in Tang et al. (2020).
- **Keep groups small and promote student engagement**—the discussion‐based nature of the course is easier with a small class, but may be possible with larger classes as well. Even for larger classes, I do not recommend forming small groups above five students, since it may be more challenging to have each student participate equally in larger groups; past literature has suggested that small groups of three to five students tend to be the most effective (Wilson, Brickman, & Brame, 2018). If in the online format, I recommend asking each student to turn on their webcam (if available) to promote engagement.
- **Rotate groups every class—**even before the transition to online teaching, I had randomized groups every class, and I continued doing so with the online format using Zoom's random breakout groups feature. Students have reported that they have been able to meet more of their peers and hear a greater diversity of perspectives than if they were placed in the same small group each class. In addition, the rotation of groups every class may increase individual accountability, a key aspect of successful group work (Wilson et al., 2018), if they will be paired with (and held accountable to) a random subset of their classmates.
- **Clarify themes and provide discussion prompts prior to small group discussion—**it can often be challenging to start a small group discussion for a particularly complex section of a paper without guidance; as such, I have found that clarifying some key themes and providing discussion prompts can facilitate the small group discussion and lead to deeper conversations and analyses of the paper. Past literature has suggested that having a clear task, such as answering and presenting on a given prompt or being tasked with leading the discussion on a given figure or section of the paper, that requires input from all students can promote cooperative learning (Tanner, Chatman, & Allen, 2003).
- **Promote equity and inclusion**—the discussion‐based nature of the class relies on students being respectful and comfortable voicing their opinions with their peers; I go through expectations and remind students of these throughout the semester. I also provide opportunities for students to provide feedback on the course, in which they are given the opportunity to alert the instructor confidentially if there are any issues with the group work. In addition, if teaching a discussion‐based course using remote instruction, it is critical that you check in on your students' technological access and their time zones and take steps to mitigate barriers, like using alternate modalities of discussion (see below).
- **Use discussion boards or other alternate modes of discussion to promote student engagement outside of class—**while my class's discussion board was not used frequently, this feature was suggested by students as a way to continue the conversation after class or to ask the instructor or their classmates questions as they read the papers. Having a discussion board also allows students in time zones where attending a synchronous discussion is not possible the opportunity to contribute.
- **Provide examples of each assignment to guide students—**the CREATE method relies on students completing assignments in a format that they may not be used to (e.g., concept mapping, figure annotating), providing examples of past student work for other papers with commentary on how they were scored can be useful for students to see the format of the assignment. Guidance should also be provided by the instructor on how to do these assignments and how they will be assessed.
- **Provide formative and summative assessments to structure student learning**—incorporating both formative and summative assessments into a class can promote student learning and allow instructors to identify misconceptions (Brazeal, Brown, & Couch, 2016). I used the assignments for each class as a means of low‐stakes formative assessment, where I would quickly skim through and grade the assignment on completion. I then allowed students to make changes and revisions to these assignments and collected them at the end of discussing the paper, where they were graded for accuracy and thoroughness. This enabled me to 1) identify key themes and misconceptions during the learning, as students were reading the paper, 2) allow more student buy‐in and lower stress since students knew that they could go back and make changes to these assignments after the in‐class discussions, and 3) assess student learning after the completion of the module by reviewing the assignments. In addition, I also incorporated a low‐stakes quiz after the paper to motivate students and serve as an additional means of assessment.

## CONFLICT OF INTEREST

None declared.

## AUTHOR CONTRIBUTIONS

**Jeremy L. Hsu:** Conceptualization (equal); writing – original draft (equal); writing – review & editing (equal).

## Data Availability

There are no pertinent data to this manuscript.
